# *qnr *Prevalence in Extended Spectrum Beta-lactamases (ESBLs) and None-ESBLs Producing* Escherichia coli* Isolated from Urinary Tract Infections in Central of Iran

**Published:** 2011

**Authors:** Iraj Pakzad, Sohbhan Ghafourian, Morovat Taherikalani, Norkhoda sadeghifard, Hamid Abtahi, Mohammad Rahbar, Neda Mansory Jamshidi

**Affiliations:** 1 *Department Microbiology, Faculty of Medicine and Clinical Microbiology Research Center, Ilam University of Medical Sciences, Ilam, Iran*; 2 *Department Microbiology, Faculty of Medicine and Molecular Medicine Research Center, Arak University of Medical Sciences, Arak, Iran*; 3 *Department Microbiology, Iranian Reference Health Laboratory, Tehran, Iran*; 4 *Department Microbiology, Karaj Islamic Azad University, Karaj/Iran*

**Keywords:** Ciprofloxacin, Iran, Resistance

## Abstract

**Objective(s):**

Extensive use of quinolones has been associated with raising level of resistance. In the current, we focused on assessing the prevalence of *Escherichia coli* resistance to quinolones and frequency of qnrA, qnrB and qnrS in non ESBLs (extended spectrum beta-lactamases) and ESBLs producing *E. coli* with blaSHV and blaTEM.

**Materials and Methods:**

One hundred and fifty *E. coli* isolates were identified during Mar. 2007 to Apr. 2008 in Milad () hospital. They were tested for ESBLs production as well as quinolone resistance. PCR was performed for detection of blaSHV and blaTEM as well as qnrA, B and S.

**Results:**

Of 150 isolates, forty-two (28%) ESBLs producing and one hundred and eight (72%) non-ESBLs producing *E. coli* were identified. 64.2% (n= 24) of *E. coli *producing ESBLs and 4.62% (n= 5) of non-ESBLs *E. coli *were resistance to ciprofloxacin. 95.2% (n= 40) and 26.1% (n= 11) of the isolates harbored blaTEM and blaSHV, respectively. 23.8% (n= 10) had both genes. 37.5% (n= 9) and 20.8% (n= 4) of ESBLs producing *E. coli* were positive for qnrA and qnrB respectively. qnrS was not identified in any isolate.

**Conclusion:**

Our study showed high frequency of ESBLs producing *E. coli* as well as quinolone resistance genes (qnrA, qnrB) in Milad hospital.

## Introduction

Low-level quinolone resistance has been associated with DNA acquired from transferable plasmids. Several studies showed a worldwide dissemination of qnr determinants among bacterial isolates. Quinolones are broad-spectrum antibacterial agents, commonly used both in human and veterinary medicine. Their extensive use has been associated with raising level of quinolone resistance. The two main mechanisms of quinolone resistance are chromosomally encoded, being either modification of the quinolone targets with changes of DNA gyrase (*gyrA*) and/or topoisomerase IV (*parC*) genes, or decreased intracellular concentration due to impermeability of the membrane or overexpression of efflux pump systems. The geographical distribution of qnrA genes is known to be wide (6), but those of the newer qnr types (qnrB (4) and qnrS (3)) have not been studied. Prior studies have not evaluated temporal changes in prevalence either.

qnrA confers resistance to quinolones such as nalidixic acid and increases MICs of fluoroquinolones up to 32-fold in *Escherichia coil*. In addition, it favors selection of associated chromosome-encoded quinolone resistance determinants that confer additional resistance to fluoroquinolones. The qnrA-like determinants have been reported worldwide from many enterobacterial species and six variants have been identified so far (qnrA1 to qnrA6). Other plasmid-mediated quinolone resistance determinants, qnrB (qnrB1 to qnrB6) and qnrS (qnrS1 and qnrS2) have been also identified in enterobacterial species, sharing 41% and 60% amino acid identity with qnrA, respectively.

Beta- lactam antimicrobial agents are the most common treatment for bacterial infections. Rates of bacterial resistance to antimicrobial agents are increasing worldwide. Production of beta-lactamases is the most common mechanism of bacterial resistance. These enzymes are numerous, and they mutate continuously in response to the heavy pressure of antibiotic use, leading to the development of extended spectrum beta-lactamases (ESBLs). The ESBL producing bacteria are typically associated with multidrug resistance, because genes with other mechanisms of resistance often reside on the same plasmid as the ESBL gene. Thus, some ESBL producing strains also show resistance to quinolones, aminoglycosides, and trimethoprim –sulfamethoxazole.

In the current study we focused on assessing the prevalence of *E. coli *resistance to quinolones and frequency of qnrA, qnrB and qnrS in ESBLs and non ESBLs producing *E. coli* with blaSHV and blaTEM in Milad Hospital (Tehran). 

## Materials and Methods

***Bacterial isolates***

One hundred and fifty *E. coli* isolates were identified during Mar. 2007 to Apr. 2008 from urinary tract infections in Milad () hospital. They were tested for ESBLs production as well as quinolone resistance.

***Detection of ESBLs producing E. coli***

The methods for the laboratory detection of ESBLs were based on recommendations of the National Committee for Clinical Laboratory Standards (NCCLS) and the Canadian External Quality Assessment Advisory Group for Antibiotic Resistance. However, we made some modifications in order to address the differences in the operations of laboratories in our settings. All the clinically significant isolates of *E. coli, *were tested against beta lactam drugs using a disc diffusion method (as advocated by the revised NCCLS interpretive criteria). Any decrease in the zone sizes for the 3rd generation cephalosporins was used as a criterion for ESBLs production (13).

**ESBL screening methods**

*Standard disc diffusion method*

*In vitro *sensitivity testing was performed using established NCCLS procedure with ceftazidim (30 μg), cefotaxime (30 μg), ceftriaxone (30 μg), aztreonam (30 μg) and cefpodoxime (30 μg). The zone diameters were read using the revised NCCLS. Any zone diameter within the “grey zone” was considered a probable ESBL producing strain requiring phenotypic confirmatory testing(14,15).

**Phenotypic confirmatory method**

Ceftazidime (30 μg) versus ceftazidime/clavulanic (30/10 μg), cefotaxime (30 μg) versus (cefotaxime /clavulanic acid (30/10 μg) and cefpodoxime versus (cefpodoxime /clavulanic acid) were placed into a Muller-Hinton agar plate lined with the test organism and incubated as described above. Regardless of the zone diameters, a > 5 mm increase in a zone diameter for an antimicrobial agent tested in combination with clavulanic acid versus its zone size when tested alone, indicated a probable ESBL production (16).

*E. coli *ATCC 25922 was used as a negative control and* Klebsiella pneumoniae *ATCC 700603 as an ESBL positive control. *K. pneumoniae *ATCC 700603 diameter ranges were as follows: cefpodoxime (10 μg) 6-9 mm, ceftazidime (30 μg) 10-18 mm, cefotaxime (30 μg) 17-25 mm, ceftriaxone (30 μg) 16-24 mm, aztreonam (30 μg) 9-17 mm.

***Quinolone resistance detection***

For detection of quinolone resistance, disk diffusion was performed as CLSI recommended by using ciprofloxacin (5 μg) disk. *E. coli* isolates which were resistant to ciprofoloxacin were suspected to harbor qnr genes (16).

***DNA extraction*** *and* ***PCR***

*E. coli *was cultured in LB broth at 37 °C 

overnight, and then DNA was extracted using the DNA extraction KIT (fermenrtase, Spain).

***PCR detection of blaTEM and blaSHV and qnr genes***

Specific primers in Table 1 were used. For blaTEM and blaSHV PCR conditions were 94 °C for 45 sec, 44 °C for 45 sec for blaTEM and 56 °C for blaSHV, and 72 °C for 60 sec, with a cycle number of 32. The PCR conditions for qnr genes were 94 °C for 45 sec, 53 °C for 45 sec, and 72 °C for 60 sec, with a cycle number of 32 (7).

## Results

Of one hundred and fifty isolates from urinary tract infections during Mar. 2007 to Apr. 2008 in Milad Hospital, forty-two (28%) *E. coli*, produced ESBLs and one hundred and eight (72%) were non-ESBLs *E. coli* isolates:

***Screening stage***

Of one hundred and fifty isolates from urinary tract infections, 69.3% (n= 104), 39.3% (n= 59), 28% (n= 42), 50.6% (n= 76) and 28% (n= 42) were resistant to ceftazidim, cefotaxime, cefpodoxime, cefteriaxone and aztreonam, respectively (Table 2). As definition, ESBLs are defined as extended-spectrum because they are able to hydrolyze a broader spectrum of beta- lactam antibiotics than the simple parent beta- lactamases from which they are derived.

Such ESBLs have also the ability to inactivate beta-lactam antibiotics containing an oxyimino-group such as oxyimino-cephalosporins (e.g.; ceftazidime, ceftriaxone, cefotaxime) as well as oxyimino-monobactam. Furthermore, they are not active against cephamycins and carbapenems. Generally, they are inhibited by beta-lactamase-inhibitors such as clavulanate and tazobactam. Any resistance to one or more of 3rd generation of cephalosporins and azteroname is suspicious for ESBLs production. In our study, forty two *E. coli* isolates were suspected to produce ESBLs.

**Table 1.** Primers used for PCR detection of blaTEM, blaSHV and qnr genes.

	Primers	Size of amplicins	Refrences
BlaSHV	F:5-AAGATCCACTATCGCCCAGCAG-3	235 bp	(13)
	R: 5-ATTCAGTTCCGTTTCCCAGCGG-3		
BlaTEM	F: 5-GAGTATCAACATTTCCGTGTC3	889 bp	(13)
	R: 5-TAATCAGTGAGGCACCTTCTC-3		
qnrA	F:5-ATTTCTCACGCCAGGATTTG	516 bp	(7)
	R: 5-GATCGGCAAAGGTTAGGTCA-3		
qnrB	F: 5-GATCGTGAAAGCCAGAAAGG-3	469 bp	(7)
	R: 5-ACGATGCCTGGTAGTTGTCC-3		
qnrS	F:5-ACGACATTCGTCAACT GCAA-3	417 bp	(7)
R: 5-TAAATTGGCACCCTGTAGGC-3

**Table 2:** Frequency of resistance of *E. coli* isolated from UTI to 3rd generation cephalosporins and monobactam.

	Ceftazidim resistance	Cefotaxime resistance	Cefpodoxime resistance	Cefteriaxone resistance	Azteronam resistance
*E. coli* isolated from UTI	104 (69.3%)	59 (39.3%)	42 (28%)	76 (50.6%)	42 (28%)

***Confirming stage***

Confirming stage was done for *E. coli* isolates suspected to produce ESBLs by ceftazidim /clavulanic acid, cefotaxime/clavulanic acid, and cefpodoxime/clavulanic acid. All the *E. coli* isolates suspected to produce ESBLs (n= 42) were confirmed by cefpodoxime/clavulanic acid. 90.4% (n= 38) and 57.1% (n= 24) were confirmed by ceftazidime/clavulanic acid, cefotaxime/clavulanic acid, respectively.

***Ciprofloxacin resistance***

All the isolates were tested for ciprofloxacin resistance. 64.2% (n= 24) of ESBLs producing *E. coli *and 4.62% (n= 5) of non-ESBLs producing *E. coli *isolates were resistance to ciprofloxacin using disk diffusion method. Thus, 19.3% (n= 29) of all isolates were resistance to ciprofloxacin.

**Figure 1.**
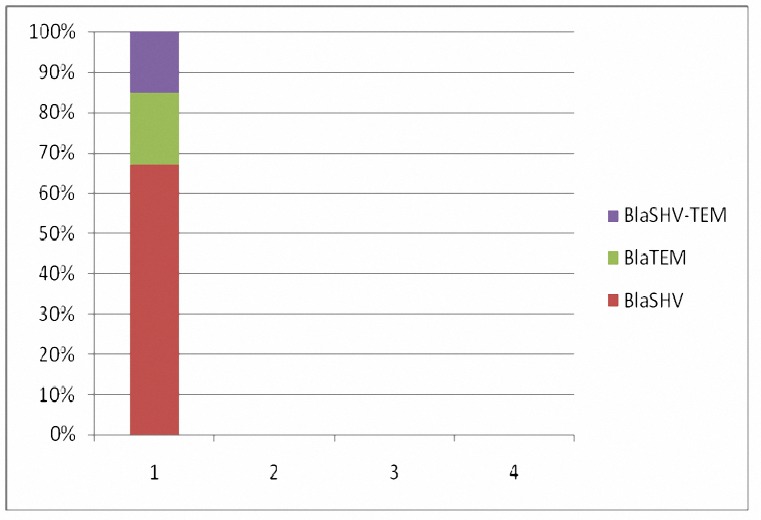
Frequency of blaSHV, blaTEM and blaSHV-blaTEM in ESBLs producing *E. coli* isolates, 95.2% (n= 40) and 26.1% (n= 11) harbored blaTEM, blaSHV, and 21.4% (n= 9) had both genes.

**Figure 2.**
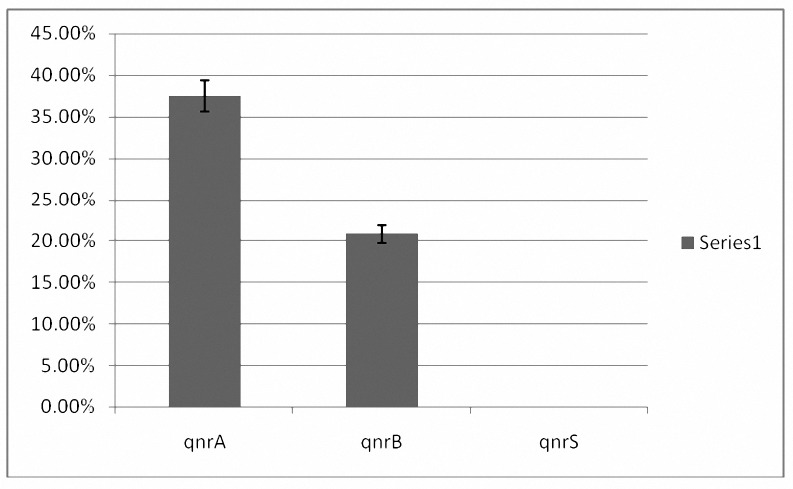
Frequency of qunr A, qnrB and qunrS in ESBLs and nono-ESBLs producing *E. coli* isolates: 37.5% (n= 9), 20.8% (n= 4) and 0% were positive for qunrA, qnrB and qunrS, respectively.

**Figure 3.**
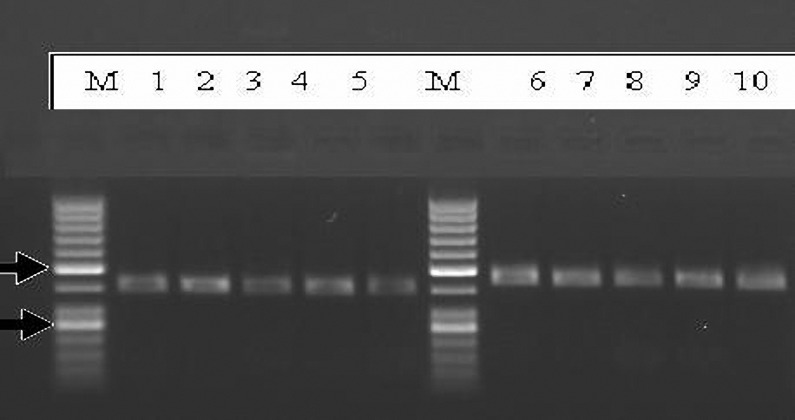
Electrophoresis of PCR product on 1% agarose gel, M (Marker 50 bp), qnrB= 469 bp (lane 1, 2, 3, 4, 5), qnrA =516 bp ( lane 6, 7, 8, 9, 10).

***PCR for detection of blaTEM and blaSHV***

Forty-two ESBLs producing *E. coli* obtained in phenotypic stage were tested for detection of blaTEM and blaSHV. Our results showed, 95.2% (n= 40) and 26.1% (n= 11) blaTEM and blaSHV harboring isolates, respectively. 21.4% (n= 9) had both genes (Figure 1).

***PCR for qnrA, qnrB and qnrS***

OF Twenty-four E. coli producing ESBLs and resistant to ciprofloxacin, all harbored blaTEM and amongst them two isolates possessed blaSHV in addition to blaTEM. 37.5% (n= 9) and 20.8% (n= 4) *E. coli* producing ESBLs (with blaTEM) were positive for qnrA and qnrB, respectively (Figure 3). No qnrS was identified in our study (Figure 3). *E. coli* with both qnrA and qnrB were found in *E. coli* producing ESBLs with both blaTEM and blaSHVgenes. Of five *E. coli* isolates that were non-ESBLs producing, only one isolate harbored qnrA (Figure 2).

## Discussion

In our study the highest antibiotic resistance occurred to ceftazidim and the lowest was to cefpodoxime and aztreonam. Interestingly, all *E. coli* suspected to produce ESBLs were confirmed by cefpodoxime/clavulanic acid. Resistance to ciprofloxacin was observed in ESBLs producing *E. coli* more than non-ESBLs producing *E. coli* isolates.

Frequency of blaTEM was higher than blaSHV. qnrA was dominant qnr followed by qnrB. *E. coli *isolates with both qnrA and qnrB were found in *E. coli* isolates with both blaTEM and blaSHV while qnrA was also found in non –ESBLs producing *E. coli* isolates. Several reports have detected a positive correlation between qnrA and the ESBLs production blaTEM and blaSHV (1, 18,19) In Chinese pediatric patients clinical isolates of ESBL or AmpC-producing *E. coli* revealed that qnr, aac(6')-Ib-cr, and ESBL-encoding genes were transferred together. qnrA-like determinants in ciprofloxacin-resistant *E. coli* isolates collected from 2000 to 2002 were estimated to be 7.7% in Shanghai, China. In Germany, qnrA-positive Enterobacter spp. and *Citrobacter freundii* isolates were detected in four patients in two intensive care units among 703 cephalosporin-resistant or fluoroquinolone-resistant Enterobacteriaceae which were tested from 34 German intensive care units from 2000 to 2003. In Korea, qnrB4 was the most frequent type in both *E. coli *and *K. pneumoniae* isolated from a tertiary care hospital. qnrB was mainly carried by *E. coli* and qnrS by *K. pneumoniae* in healthy children in Peru and Bolivia. In close association of qnr with aac(6')-Ib and aac(6')-IIc in clinical isolates of *E. coli* and *K. oxytoca* producing ESBL or MBL was noticed. In clinical isolates of *E. coli* only qnrS was identified from Japan. qnrA determinants were found in up to 48% of VEB-1-positive enterobacterial isolates from Bangkok, Thailand, qnrB determinants were associated with the ESBL SHV-12 in several isolates and 62% of ESBLs production of *E. coli* were resistance to ciprofloxacin. Our results also showed high resistance to ciprofloxacin which was concordant with the above-mentioned reports. Our study also showed that some of *E. coli* isolates (ESBLs and non-ESBLs producing) didn’t have qnr genes but were resistant to ciprofloxacin. This indicted other resistance mechanisms such as changes of DNA gyrase (*gyrA*) and/or topoisomerase IV (*parC*) genes, or decreased intracellular concentration due to impermeability of the membrane or overexpression of efflux pump systems. In this study, high frequency of quinolone resistance genes (qnrA, qnrB) may be due to fact that all isolates were originated from one hospital. In addition, environmental conditions and the antibiotic burden may affect the frequency of quinolone resistance.

The clinical relevance of the multidrug resistance among ESBL-producing *E. coli *isolates is of great concern due to the severely limited therapeutic options and increased risk of treatment failure in patients infected with such strains.

Since plasmids frequently carry both the ESBL and aminoglycoside resistance genes and many Enterobacteriacea species have also chromosomal resistance to quinolones, the ESBL-producing Enterobacteriacea are commonly multidrug resistant. Association of antibiotic resistance genes may explain in part the frequent association between fluoroquinolone and expanded spectrum cephalosporin resistance in *E. coli*. In addition, it raises the issue of the nature of antibiotic molecules that may select this co-resistance. We do not know if there is a special link between the two emerging mechanisms of resistance in* E. coli* plasmid-mediated quinolone resistance and ESBL in community-acquired pathogens. This was first report of qnrA, B in *E. coli* producing ESBLs and undetectable qnrS in .

## Conclusion

Our study showed that frequency of blaTEM was higher than blaSHV in ESBLs producing *E. coli *isolates, and also quinolone resistance genes qnrA was dominant qnr followed by qnrB.
